# Immunological tolerance and tumor rejection in embryo-aggregated chimeric mice – Lessons for tumor immunity

**DOI:** 10.1186/1471-2407-8-370

**Published:** 2008-12-16

**Authors:** Alexander Y Wagner, Eric Holle, Lori Holle, Xianzhong Yu, Günter Schwamberger

**Affiliations:** 1 The Oncology Research Institute, Greenville Hospital System University Medical Center, 800 West Faris Road, Greenville, SC 29605, USA; 2 Christ Church Episcopal School, 245 Cavalier Drive, Greenville SC 29607, USA; 3 Dept. of Biological Sciences, Clemson University, Clemson SC 29634, USA; 4 Dept. of Molecular Biology, University of Salzburg, Hellbrunner Str. 34, A-5020 Salzburg, Austria

## Abstract

**Background:**

Rejection of transplanted tumors by the immune system is a rare event in syngeneic hosts, and is considered to be dependent on the local interaction of defensive immune reactions and tumor tolerance mechanisms. Here, we have enlisted the aid of a unique set of embryo-aggregated lineage chimeric mice derived from C57/BL6 and FVB donors to study the interplay between local and systemic tumor immunity and tolerance in rejection of mouse B16 melanoma cells, syngeneic to the C57/BL6 donor strain.

**Methods:**

Two variants of embryo-aggregated chimeric mice with either variable or no contribution of C57-derived cells to their skin were generated by the fusion of different ratios of morula stage blastomers. Chimeric mice were analyzed for s.c. growth of B16 tumors in comparison to their respective donor strains as well as normal F1 hybrids, and the relative frequencies of cellular components of the immune system by FACS analysis of peripheral blood or lymph node cells.

**Results:**

B16 tumors grew significantly faster in mice with full chimerism in their skin as compared to syngeneic C57 or semi-syngeneic C57 × FVB F1 hosts. In contrast, s.c. tumor growth was either absent or significantly reduced in chimeric mice lacking C57-derived cells in their skin, but tolerant to C57 tissue in other organs. Comparison of the relative frequencies of various immune cells in the periphery via FACS-analysis did not reveal any significant differences between the two types of chimeric mice with respect to their donor strains.

**Conclusion:**

Our data suggest a complex interplay between mechanisms of local peripheral tolerance and innate antitumor mechanisms possibly involving NK cell allorecognition as a basis for the differential growth or rejection of B16 tumors in these unique chimeric mice, which we suggest to constitute a valuable new model system for the study of immune-mediated tumor rejection.

## Background

While recent advances in tumor immunology have led to the establishment of methods of generating significant clinical anti-tumor immune responses, outright tumor rejection has proved elusive in most cases. Therefore, an understanding of the distinct difference between an anti-tumor immune response and outright tumor rejection remains an important goal within the field of tumor immunology. B16 melanoma tumors in the C57/6J mouse strain represent one of the most commonly studied murine models for tumor immunotherapy. It is well known that the B16 cell line will grow and quickly establish tumors when injected s.c. or i.v. in syngeneic C57BL/6J mice. Discrete tumor rejection in this system would thus serve as a good model for effective immunotherapy.

Tumor rejection in syngeneic mice is believed to be the consequence of recognition of discrete tumor antigens by the host's immune system, predominantly via recognition of mutated or over-expressed "self" peptides displayed in the context of MHC-I molecules by cytotoxic (CD8^+^) T lymphocytes [1]. On the other hand, as shown in a series of elegant studies by North and colleagues in the 1980's [2], establishment of a tumor-specific cytotoxic T cell response per se usually is not sufficient to eradicate transplanted tumors or restrict their growth. This state of "concomitant" tumor immunity has been attributed to tumor tolerance via active immune suppression mechanisms exerted by tumor-specific suppressor T cells [3,4]. This concept has been revived in recent years by the discovery of so-called regulatory T cells (T_reg_) as crucial elements of immunologic self tolerance [5] but also of induced tolerance to growing tumors [6]. Thus paradoxically, tumors may grow and eventually kill their hosts despite systemic antitumor immunity, due to localized tumor-antigen-specific suppression of the co-existing protective immune response.

In accordance with the principles of transplantation immunology, transplanted tumors usually grow just as well in semi-syngeneic F1 offspring of their syngeneic hosts as in the latter, whereas the same tumor is rapidly rejected by the allogeneic counterpart as a consequence of allorecognition of the tumor cell's MHC/peptide complexes [7], representing the two extremes of tumor tolerance vs tumor rejection. Apart from semi-syngeneic mice, embryo-aggregated chimeric mice (not to be confused with "hematopoietic chimeras" generated by bone marrow transplantation) will also harbor the same genetic background derived from both donor strains, albeit in still distinct cells dispersed throughout the body, and thus may serve as an attractive alternative model for tumor immunity. Aggregation of the blastomers from morula stage embryos of two disparate mouse lines results in the formation of a chimeric morula that develops into a mouse that contains cells from the two distinct parental backgrounds [8]. In this manner it is possible to generate mice that contain a mixture of cells from two allogeneic mouse lines. The immune system of these chimeric mice therefore should and in fact has been shown to be tolerant to all cell types present within the chimeric mouse [9], which is consistent with the normal health status of these mice.

While such embryo-aggregated chimeric mice have been produced by and known to embryologists for several decades [10,11], a unique class of these chimeric mice have been generated for this study that have not been previously reported. In addition to full chimeric mice, chimeric mice devoid of one of the two allogeneic parental backgrounds within their skin have been generated to study the role of the local genetic environment on tumor tolerance vs tumor rejection. These unique chimeric mice have contributions from the C57 strain in earlier developing tissues that derive from the mesendoderm, precursor of the endoderm and mesoderm, (tissues derived from the mesoderm include the heart and blood as well as others while other internal organs such as lung, liver, digestive system and thymus [12] derive from the endoderm) in spite of the decreased number of C57 blastomers present in the developing chimeric embryo but lack contributions from this strain in ectoderm derived skin. While some studies on the susceptibility of chimeric mice to spontaneous tumor development have been performed (reviewed in [13]), to our knowledge, this report is the first attempt to use such mice as a system to study tumor immunology with a defined transplantable tumor model. By comparing s.c. B16 tumor growth in syngeneic C57BL/6J mice, allogeneic FVB/NJ mice, semi-syngeneic C57 × FVB F1 mice, fully chimeric C57/FVB mice and C57/FVB chimeric mice devoid of C57 skin, a complex interplay of local immune tolerance and defense mechanisms in distinct tumor rejection is suggested.

## Methods

### Mice

FVB/NJ and C57/BL6J mice were from Jackson Laboratories (Bar Harbor, ME) and CD1 mice were from Charles River Laboratories (Willmington, MA). All mice were housed in the Oncology Research Institute's animal facility and were pathogen free. Experiments performed were in compliance with the guiding principles of the *Guide for the Care and Use of Laboratory Animals *(available at ) and approved before use by the Institutional Animal Care and Use Committee of the Greenville Hospital System University Medical Center.

### Collection of embryos

Donor FVB/NJ (FVB) and C57BL/6J (C57) female mice were superovulated with i.p. injections of 5 IU of Pregnant Mare Serum Gonadotropin (Calbiochem; Gibbstown, NJ), followed 48 h later by i.p. injections of 5 IU of human chorionic gonadotropin (Sigma Chemical Company; St. Louis, MO). The donor mice were mated to FVB and C57 stud males. 46 h after mating, donor females showing vaginal plugs were sacrificed, and 2-cell embryos were flushed from the oviducts using a 30 G 1/2 inch needle attached to a 3 ml syringe filled with M2 medium. The embryos were washed several times in 75 μl micro drops of M16 medium and cultured 24 h to 8-cell in 50 μl micro drops of M16. All micro drops used for embryo culture and manipulation were covered with mineral oil (Sigma Chemical Company; St. Louis, MO) in 60 mm petri dishes. Dishes with M16 medium were kept at 37°C in a 5% CO_2_/air mixture, and dishes with M2 medium were kept at 37°C in air. M2 and M16 media were made in-house.

### Preparation of embryos

The zona pellucidas were removed from the FVB and C57 8-cell embryos by placing them into 100 μl micro drops of a 0.5% protease (Sigma Chemical Company; St. Louis, MO) in M2 solution for 10 min at 37°C. The zona free embryos were washed by passage through several 75 μl micro drops of M16 and placed in M16 holding drops. Individual C57 blastomeres were generated from C57 embryos by placing them into 100 μl micro drops of Mg^++ ^and Ca^++ ^free Dulbecco's PBS (Gibco; Carlsbad, CA) plus 0.6% BSA (Sigma Chemical Company; St. Louis, MO) for 15 to 20 min at room temperature. Separation of the blastomeres was aided by gentle pipetting with a flame polished embryo transfer pipette.

### Aggregation of embryos

Aggregation plates were prepared by placing multiple 25 μl M16 drops covered with mineral oil in 60 mm petri dishes and concave depression wells were made in the center of each M16 drop by firmly pressing an aggregation needle (BLS; Hungary) into the plastic. The plates were kept at 37°C in 5% CO_2 _in air. FVB 8-cell embryos were paired with 8-cell C57 embryos in the depression wells. FVB 8-cell embryos were also paired in various combinations with individual C57 blastomeres at 1:8, 2:8, 3:8, and 4:8 (C57: FVB) ratios in the depression wells to attempt to generate chimeras with some wholly FVB tissues. The aggregated embryos were cultured at 37°C in 5% CO_2 _in air for 24 h until they developed into early blastocysts. Pseudopregnant CD1 (Charles River Laboratories, Wilmington, MA) female recipient mice at 0.5 and 2.5 days post conception (dpc) were anesthetized with a 1.4% avertin solution in PBS (0.1 ml/5 g of body weight). 14 to 20 aggregated blastocysts were transferred into the oviducts of each 0.5 dpc recipient mouse, and the uterine horns of each 2.5 dpc mouse.

### Generation of chimeric mice

255 aggregates were made and transferred into 15 CD1 recipient females. 66 pups were born or taken by cesarean section. 52 pups survived. Nine mice had varying ratios of black and white coat color, and ten were later identified as white coat chimeras by flow cytometric analysis of their blood lymphocytes.

### Tumor cell culture

B16-F0 murine melanoma cells were obtained from ATCC (Manassas, VA) and cultured in DMEM (Fisher; Pittsburgh, PA) modified with 10% FBS (Fisher). Cells were cultured at 37°C in the presence of 5% CO_2_. Cells were harvested by trypsinization and washed in PBS prior to use for tumor inoculation.

### Mouse tumor challenge

Five C57BL/6J, five FVB/NJ, five C57BL/6J × FVB/NJ F1 hybrids (C57 × FVB), six C57/FVB chimeras with black and white segmented coat color and five C57/FVB chimeras with pure white coat color were injected intradermally with 1 × 10^6 ^B16-F0 cells. All mice were inoculated with B16-F0 tumor cells between 40 and 50 days of age. At day 18 post tumor cell inoculation mice with tumors estimated to weigh more than 1 g were sacrificed by CO_2 _asphyxiation and mice with smaller tumors were anesthetized with a 1.4% avertin solution in PBS (0.1 ml/5 g body weight) and the tumors excised and weighed. Following removal of the smaller tumors from anesthetized mice the small incision remaining after tumor excision was closed using a sterile wound clip.

### Determination of H-2K^b ^(C57) and H-2K^q ^(FVB) leukocyte percentages in mice

In order to determine the haplotype (H-2K^b^/H-2K^q^) percentage ratio of white blood cells in control and chimera mice, white blood cells were isolated from mouse whole blood, stained with the cognate fluorescent antibodies and analyzed by flow cytometery. Blood was collected from the tails of mice by use of a heparin coated blood collection tube, Fisher (Pittsburgh, PA) and 0.5 M EDTA added to prevent coagulation. Approximately 100 μl of blood was collected from each mouse, 2 ml of 1 × FACSlyse buffer (BD Biosciences; Hayward, CA) added to lyse the red blood cells and the suspension was incubated at RT for approximately 10 min. Samples were then centrifuged at 300 × *g *for 5 min at room temperature and washed twice with PBS.

Approximately 1 million white blood cells were resuspended in staining buffer (1% FBS, 0.1% sodium azide, PBS) for each reaction and then 1 μg of the appropriate antibody was added. Both H-2K^b ^(clone AF6-88.5) and H-2K^q ^(clone KH114) antibodies were FITC conjugated and obtained from BD Biosciences (San Jose, CA). Cells were incubated with antibody for 30 min on ice in the dark and then washed twice with staining buffer. Cells were resuspended in 250 μl of staining buffer and then analyzed. The GUAVA EasyCyte obtained from GUAVA technologies (Hayward, CA) was used for flow cytometric analysis. Various leukocyte populations were gated and 10,000 events were analyzed using the GUAVA Express Plus program. Data was then analyzed by use of CytoSoft 3.6.1 software.

### Analysis of the percentage CD4^+^, CD8^+^, and CD45/B220^+ ^lymphocytes in control and chimeric mouse blood

In order to analyze the percentage of CD4^+^, CD8^+^, and CD45/B220^+ ^lymphocytes in the blood of control and chimeric mice, 50 μl of whole blood was collected via a heparin coated bleeding tube and added to 1 ml of wash buffer (1% FBS, 0.1% sodium azide, PBS). Cells were then pelleted by centrifuging at 500 × *g *for 5 min. All supernatant was removed from the cell pellet except for approximately 200 μl. 10 μg of rat IgG (BD biosciences; San Jose, CA) was then added and each sample incubated on ice for 15 min. Rat IgG was added to the samples to block nonspecific antibody binding to the cells. 0.5 μg each of anti CD4, CD8, and CD45/B220 antibodies were added to each sample and the mixture then incubated for 30 min on ice in the dark. All antibodies were obtained from BD Biosciences and anti-CD4 was PE-conjugated, anti-CD8 was FITC-conjugated, and anti-CD45/B220 was PE-Cy5-conjugated. Following antibody incubation, cells were washed once with wash buffer and then 1 ml of ACK lysing buffer (Cambrex; East Rutherford, NJ) was added to lyse the red blood cells. Samples were incubated for 5 min in ACK lysing buffer at RT. Samples were then washed once with wash buffer and resuspended in 300 μl of wash buffer. Cells were analyzed using the BD FACS Calibur and data analyzed using the Cell Quest program.

### Analysis of the percentage CD4^+^CD25^+^Foxp3^+ ^T_reg _cells in control and chimeric mouse lymph nodes

Lymph nodes were obtained from C57, FVB, C57FVB F1 hybrids, C57/FVB chimeras with black and white segmented coat color and C57/FVB chimeras with pure white coat color and single cell suspensions in FACS buffer (phosphate-buffered saline containing 0.1% sodium azide and 0.1% bovine serum albumin) were obtained by pressing tissues through a 40 μm Nylon Cell Strainer (BD Biosciences Discovery Labware, Bedford, MA) with a syringe plunger (BD Biosciences Discovery Labware, Dedford, MA). After centrifugation at 1500 rpm for 5 min, the cell pellet was resuspended in ACK lysing buffer (Lonza, Walkersville, MD) for 5 min to lyse red blood cells. The cells were then washed with FACS buffer twice and counted. 1 × 10^6 ^cells in 50 μl FACS buffer were used for each staining. After the cells were incubated with 10 μg/sample rat IgG (Zymed Laboratories, South San Francisco, CA) on ice for 15 min, 1 μg/sample of FITC-labeled rat anti-mouse CD4 and PE labeled rat anti-mouse CD25 (BD Biosciences Pharmingen, San Jose, CA) were added respectively and incubated on ice for another 30 min. The cells were further washed with FACS buffer. After the cells had been stained with CD4-FITC and CD25-PE and washed they were fixed and permeabilized using a Fixation/Permeabilization kit (BD Bioscience, San Jose, CA) and then incubated with PE-Cy5 labeled rat anti-mouse Foxp3 antibody (eBioscience, San Diego, CA) for 30 min, washed with the Perm/Washing buffer provided with the kit, resuspended in FACS buffer and analyzed on the BD FACSCalibur. The FACS data was analyzed using the Cell Quest program.

### Statistical analysis

Statistical relevance of experimental data was assessed by the Student's *t*-test.

## Results

### Growth of B16 tumor cells in C57/FVB chimeric mice

**Figure 1:**
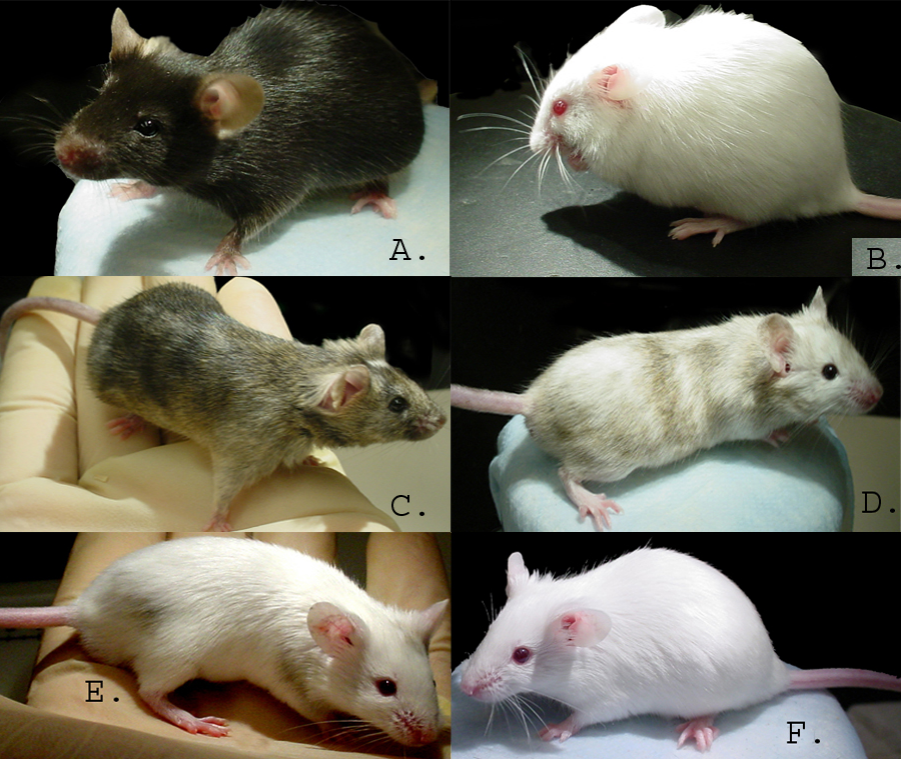
**Images of several mouse chimeras generated by fusion of C57 8-cell and C57 blastomers with FVB 8-cell embryos**. A) typical C57/6J mouse, B) a typical FVB/NJ mouse, C) mouse # 2, a C57/FVB chimera produced by the fusion of FVB and C57 8-cell embryos with black and white segmented coat color, D) mouse #4 another C57/FVB chimera produced by the fusion of FVB and C57 8-cell embryos with black and white segmented coat color, E) mouse #6 another C57/FVB chimera produced by the fusion of FVB and C57 8-cell embryos with minimal black and predominate white segmented coat color and F) Mouse #8 a C57/FVB chimera produced by fusion of 2 C57 blastomers with an FVB 8-cell embryo with pure white coat color.

**Figure 2:**
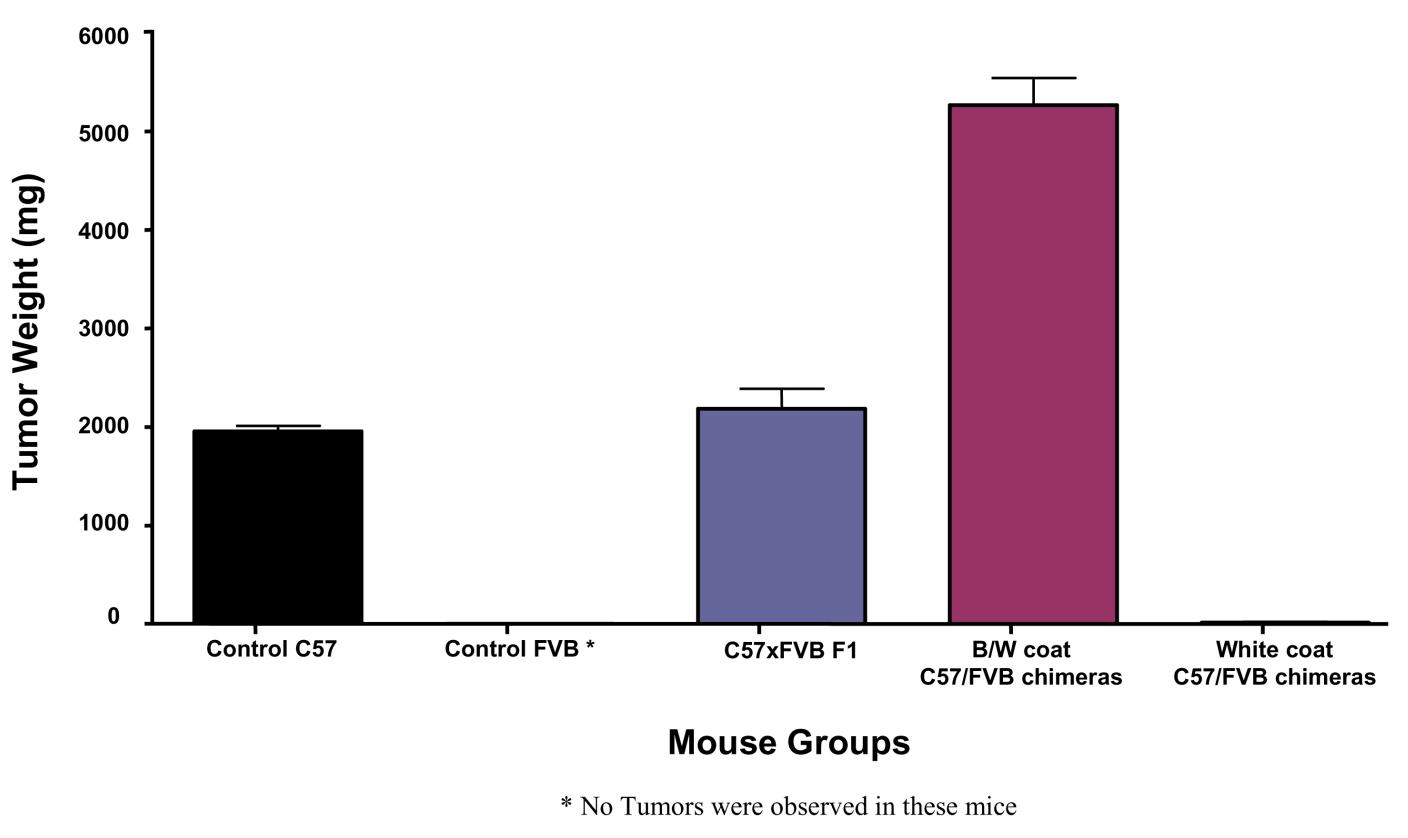
**Tumor weights in normal and chimeric mice at day 18 following intradermal inoculation with 1 × 10^6 ^B16-F0 tumor cells**. Each group includes 5 mice. The B/W coat chimera group includes mice # 1–5 from Table 1 and the White coat chimera group includes mice # 7–11 from Table 1. Error bars represent standard deviations of samples from individual mice. Differences in tumor weight of the B/W coat chimera group and the W coat chimera group with respect to the C57 × FVB F1 control group are highly significant (p < 0.001 for both sets).

Embryo-aggregated chimeric mice with different levels of chimerism between C57 and FVB tissues were generated by varying the ratio of C57 blastomers with respect to FVB 8 cell embryos. Variations in chimerism were especially notable in the contribution of C57 to the skin of chimeric mice, ranging from black and white segmented over minor patches of black to pure white (FVB) coat colour (Fig. 1). Yet, even the latter showed an, albeit reduced, level of chimerism with respect to their blood cells (Table 1). All types of chimeras were healthy and did not show any evidence of an abnormal immune status in terms of enhanced sensitivity to spontaneous infections or autoimmune disease. Nevertheless, individual chimeric mice showed dramatic differences in terms of growth or rejection of s.c. injected B16 melanoma cells of C57 origin, which was clearly correlated with the level of skin chimerism (Table 1). Whereas B16 tumors grew rapidly in mice with substantial coat chimerism (termed fully chimeric mice herein), tumor growth in mice with overly white, FVB-derived skin (termed partial chimeras herein) was either marginal, yielding minute, largely necrotic tumors, or not observed at all. Fig. 2 gives a quantitative comparison of B16 tumor growth in these two types of chimeric mice with growth of the same tumor in either allogeneic FVB mice, the syngeneic host C57 or C57 × FVB F1 as the control most closely matching the genetic setup of the chimeras. Evidently and as expected, FVB mice fully rejected the tumor transplant, whereas there was no difference in tumor growth between the syngeneic C57 host and the semi-syngeneic C57 × FVB F1. Quite remarkably however, as compared to these controls, tumor growth was either significantly enhanced or suppressed in the two types of chimeric mice. Thus, the level of skin chimerism with respect to C57 derived tissue exerted a marked influence on the growth or rejection of the C57-derived tumor in otherwise chimeric mice. Reduced tumor size in partial chimeras was not due to delayed rejection of tumors initially growing normally, but reflects an immediate type of tumor rejection or growth suppression. Interestingly however, two of the partially chimeric mice (mice #8 and #10) that rejected the primary s.c. tumor inoculum developed a quite massive tumor in the peritoneal cavity 6 – 8 weeks later, demonstrating that these mice are not generally rejecting these B16 tumors, but effectively do so in their skin.

**Table 1:** Relative chimerism of individual mice versus tumor progression

**C57/FVB Mouse Chimera**	**Coat Chimerism**	Hematopoietic Chimerism H2K^b^/H2K^q ^(% Lymphocytes)*	**B16-F0 Tumor Weight at Day 18 Post Injection**
#1	C57/FVB	56/44	5478 mg^a^
#2	C57/FVB	52/48	5802 mg^a^
#3	C57/FVB	42/58	4310 mg^a^
#4	C57/FVB	57/43	5021 mg^a^
#5	C57/FVB	51/49	5724 mg^a^
#6	C57/FVB**	36/64	16 mg^b^
#7	Only FVB	9/91	No Tumor
#8	Only FVB	7/93	No Tumor^c^
#9	Only FVB	7/93	24 mg^b^
#10	Only FVB	5/95	14 mg^b,c^
#11	Only FVB	8/92	31 mg^b^

*- Haplotype frequencies determined for whole blood lymphocytes

**- The black (C57) coat color contribution was minimal. See Figure 2.

^a^- Mouse was sacrificed at day 18.

^b^- Tumor was surgically removed on day 18 and the mouse continued to be monitored. Resected tumors were largely necrotic at time of resection.

^c^- Died of a large peritoneal tumor 8 weeks following s.c. tumor cell injection

### Immune system of chimeric mice

**Figure 3:**
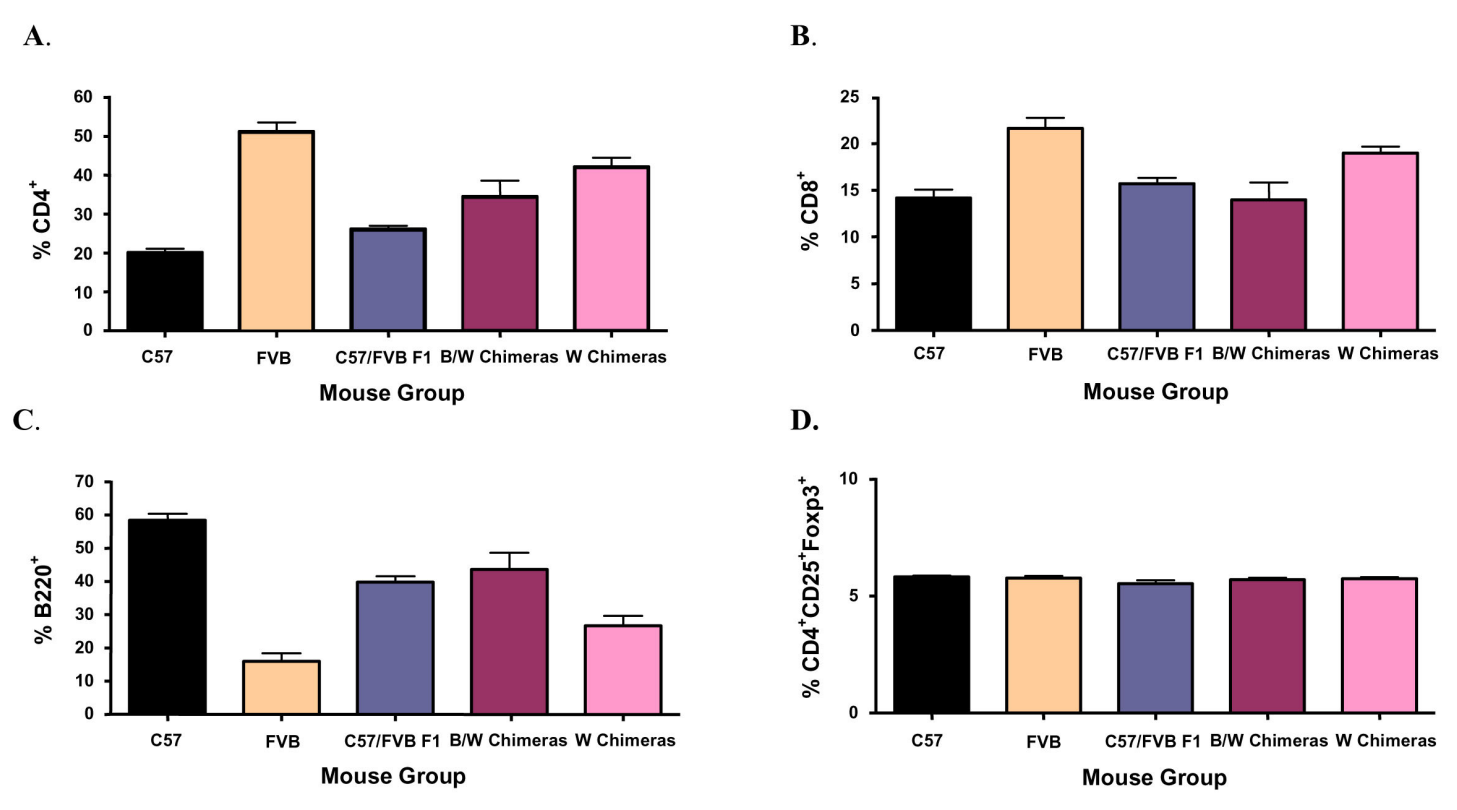
**Percentage of CD4^+ ^T cells, CD8^+ ^T cells, B220^+ ^B cells and CD4^+^CD25^+^Foxp3^+ ^T_reg _cells in normal and chimeric mice**. A) Percentage of CD4^+ ^T cells per total blood lymphocytes. Each group includes 3 mice 30–40 days of age. The B/W chimera group includes mice # 3–5 from Table 1 and the W chimera group includes mice # 9–11 from Table 1. B) Percentage of CD8^+ ^T cells per total blood lymphocytes. Each group includes 3 mice 30 – 40 days of age. The B/W chimera group includes mice # 3 – 5 from Table 1 and the W chimera group includes mice # 9 – 11 from Table 1. C) Percentage of B220^+ ^B cells per total blood lymphocytes. Each group includes 3 mice 30 – 40 days of age. The B/W chimera group includes mice # 3 – 5 from Table 1 and the W chimera group includes mice # 9 – 11 from Table 1. D) Percentage of CD4^+^CD25^+^Foxp3^+ ^T_reg _cells per total lymph node lymphocytes. Each group includes 4 mice 30 – 40 days of age. The B/W and W chimera groups include mice different from those described in Table 1 since it was necessary to sacrifice these mice to recover their lymph nodes.

These quite unexpected differences in tumor growth or rejection prompted us to analyze the relative frequencies of various cells of the adaptive immune system in these chimeric mice in comparison to the respective "parental" controls or the F1 offspring. However, apart from a marked expansion of the T cell over the B cell compartment in FVB vs C57 donor strains, no significant differences between these groups of mice in terms of total blood T and B lymphocytes, and notably also CD4^+^CD25^+^Foxp3^+ ^T_reg _cells in lymph nodes were observed (Fig. 3). Haplotype frequencies of individual lymphocyte subsets were also tested for some chimeric mice, but did not reveal significant differences from the respective frequencies of total lymphocytes (data not shown), suggesting that there is no specific haplotype bias for individual lymphocyte subsets in chimeric mice. The same is also true for the numbers and haplotype frequencies of predominant cells of the innate immune system (neutrophils and blood monocytes (data not shown), demonstrating that there is no gross alteration in the basic constituents of the immune system in these chimeric mice, which is in line with their normal overall health status.

## Discussion

Because the observations and results described herein derive from the first study of the development of a defined transplantable tumor in embryo-aggregated chimeric mice, more questions than answers may have emerged. Nonetheless, two predominant observations are that B16 tumors grow significantly faster in full chimeric mice than in their syngeneic hosts or C57 × FVB F1 offspring and that these same B16 tumors are either fully or partly rejected within the skin of chimeric mice with a non-chimeric skin allogeneic to the tumor implant, although these unique chimeras are tolerant toward C57-derived tissue in other organs. Clearly, established and accepted precepts of present day immunology would not predict either the enhanced growth of B16 tumors in fully chimeric C57/FVB mice or the rejection of these same tumors in the same partially chimeric mice since both of these hosts should be tolerant for the MHC class b haplotype of these tumor cells, as suggested by early transplantation studies with chimeric mice, termed "allophenic" by these authors [9]. While at first glance the results reported in their paper would also imply a selective rejection of "mismatched" donor normal skin transplants by single color-coated "allophenic" recipient mice, no evidence for actual chimerism of these mice was presented. Since it is well established that in vitro aggregation of blastomers from two different donors quite often does not result in the generation of detectable chimerism [11] (A. K. Tarkowski, personal communication), these "allophenic" mice, unlike the ones reported here with proven chimerism, rather have to be considered as essentially non-chimeric. In fact, a later study of the same group [14] failed to demonstrate chimerism in various tissues of the vast majority of such single color-coated "allophenic" mice, suggesting the observed rejection of unmatched donor skin to be due to classical allograft rejection, and thus to be mechanistically unrelated to the findings presented here.

While at the current status of experimental work the following discussion has to remain largely speculative, the puzzling data obtained may justify a closer look at the current concepts of allorecognition, tolerance and tumor immunity, in search for possible explanations for the enhanced growth vs rejection of the B16 tumors in these two variants of chimeric mice.

The concept of immunological tolerance is based on two basically independent mechanisms, termed central and peripheral tolerance, respectively, leading to clonal deletion and/or functional inactivation of autoreactive lymphocytes [15]. Since rejection of tissue transplants is known to be mainly based on alloreactive T cells [16], we will limit our further discussion on mechanisms operative in T cell tolerance. Central tolerance to self tissue antigens, considered to be the predominant mode of tolerance [17,18], of both CD4^+ ^and CD8^+ ^T cells is acquired in the thymus by clonal deletion of T cells reactive to self antigens displayed by epithelial cells and/or dendritic cells (DC) in the thymic medulla during T cell maturation [19]. Whereas clonal deletion of CD4^+ ^T cells seems to be primarily dependent on presentation of endocytosed structures displayed in the thymus by thymic DC's, central tolerance at the level of CD8^+ ^T cells is established predominantly via presentation of internal self epitopes on the MHC-I molecules on thymic epithelial cells, although thymic DC's may also participate in this reaction [20]. Wide coverage of self structures is ensured by the random expression of peripheral tissue antigens under the control of the autoimmune regulator (AIRE) element and other less well characterized mechanisms [21,22]. Peripheral tolerance, considered as a polishing mechanism for autoreactive clones that have escaped the mechanisms of central tolerance or clones directed to tissue specific self peptides not displayed in the thymus [23], is based on several independent mechanisms, clonal anergy, activation-induced cell death (AICD) [23] and the generation of antigen-specific CD4^+ ^and CD8^+ ^T_reg _cells [24]. The common aspect of these mechanisms is antigen recognition in lack of appropriate costimulatory signals or by specific tolerogenic/polarizing signals provided by quiescent antigen presenting cells in peripheral tissues [25-27]. Whereas anergy and AICD may be viewed as a continuation of clonal deletion of functional T-cells in the periphery without direct impact on the local immunological environment, T_reg _cells exert their effect by local suppression of immune reactions either via deletion or functional inactivation of activated immune cells or via inhibitory cytokines like TGF-β, IL-10 or IL-35 [28], thus dominantly affecting immunity locally in response to specific antigens, albeit lacking antigen specificity.

In a chimeric thymus, central tolerance to all types of self antigens should be established, including the MHC molecules of both "parental" strains just as in normal F1 offspring. However, there may be one fundamental difference between F1 offspring and chimeras in terms of antigen presentation via MHC-I molecules, which may necessitate enhanced peripheral tolerance of the CD8^+ ^T cell compartment. In F1 offspring, due to heterozygosity, self peptides derived from either one or the other strain will be more or less randomly presented by MHC-I molecules of both strains, resulting in a larger variation of potential self structures displayed in the thymus. In chimeras, on the other hand, self peptides derived from either donor strain will be predominantly presented in the context of the donor-specific MHC-I molecules on thymic epithelial cells. As a consequence, CD8^+ ^T cell clones reactive to peptides of strain A in the context of MHC-I of strain B may not be deleted at an equal efficiency as "matched pairs", resulting in a larger number of surviving clones with potential autoreactivity, although these clones should not be able to directly attack normal tissue cells due to classical MHC restriction [29]. Conversely, as pointed out above, since tolerance induction for CD4^+ ^T cells is primarily based on presentation of endocytosed "external" structures and thus by definition is promiscuous with respect to peptides and MHC-II molecules of both donors, no differences would be expected at the level of central tolerance of CD4^+ ^T cells or CD4^+ ^T_reg _cells, which is in agreement with our findings.

Further maturation of effector T cells from antigenically predetermined naïve T cells is dependent on antigen presentation by DC's [30]. With respect to MHC-I restricted CD8^+ ^T cells, stimulation of naïve T cell clones by tissue antigens requires so-called "cross presentation" of structures picked up in the periphery from dead cells on the MHC-I molecules of DC's [31]. Since in chimeric mice DC's should pick up material from dead cells irrespective of their descent, self peptides of strain A should now also show up on the MHC-I molecule of strain B and vice versa. But in lack of appropriate "danger signals" and thus costimulation [32], this should again result in the induction of peripheral "cross tolerance" [33]. In contrast to normal F1 offspring however, in chimeric mice, there should still be plenty of potentially reactive CD8^+ ^clones available, due to lacking clonal deletion in the thymus. Consequently, this should also give rise to a larger number of CD8^+ ^but not CD4^+ ^T_reg _clones that in turn should serve to dampen local immune responses, and thus may explain enhanced tumor growth in the fully chimeric skin or the chimeric organs of the partial chimeras. As this should represent a local phenomenon, driven by cross presentation of tissue antigens, the extent of this effect should at least be markedly lower in non-chimeric tissues. Interestingly, this notion may get support from early studies on immune tolerance mechanisms in embryo-aggregated chimeric mice [34], demonstrating low but significant in vitro cytotoxicity of isolated lymph node cells from chimeric mice against normal donor fibroblasts which however is specifically suppressed by a soluble factor in the sera of these mice. Although performed well before the era of modern T cell immunology, these experiments would argue for some active suppression mechanism of potentially "autoreactive" cells in these chimeric mice that have escaped central tolerance mechanisms and thus would be in accordance with the concept outlined above.

However, this hypothesis cannot explain the enhanced tumor rejection observed in the non-chimeric skin of partial chimeras compared to the syngeneic host. If, as classic immunology would suggest, tumor cells are rejected by allogeneic hosts as a result of either direct or indirect allorecognition of the foreign MHC/peptide complexes by the host's T cell receptors [35,36] then such allorecognition should not occur within a chimeric mouse co-tolerant for the MHC molecules of both its tissue types and cells of either of these tissue types should not be rejected. While this is indeed the case with the fully chimeric mice, rejection or partial rejection is clearly observed in hosts tolerant of the tumor cell's MHC haplotype in the chimeric mice with a non-chimeric skin. Since this rejection by definition cannot be the result of classical allorecognition we have to look elsewhere for its origin. The most obvious explanation would be that these mice lack tolerance specifically to some C57 skin antigens to which their immune system was never exposed and thus mount a normal immune response to these structures. Current concepts of central tolerance would however predict random expression of various tissue antigens in the thymus [21,22]. Thus, even in all-white skin partial chimeras, skin-specific antigenic peptides of both donor strains should be expressed in the thymus. As a consequence, there should be no difference in central tolerance to these structures between full and partial chimeras. But given the lower overall percentage of chimerism in the latter, it may be argued that some C57 skin antigens may have slipped from central tolerance induction in these mice. However, three lines of experimental evidence argue against this scenario. First, chimeric mice which do have small patches of C57-derived skin (mouse #6) and thus must be tolerant to C57 skin antigens nevertheless rejected the s.c. B16 tumor implants to the same extent as pure white skin chimeras. Second, delayed growth of a large secondary tumor in the peritoneal cavity of two of the latter (mice #8 and #10) that had rejected the B16 tumor cells within their skin demonstrates that these mice were not immune to the tumors in their chimeric tissues, even though the number of tumor cells that may have escaped to the peritoneal cavity must have been only a small fraction of the cells inoculated s.c., probably also reflecting the suppressive environment in chimeric tissues discussed above. In addition, this further argues against a conventional alloresponse as the basis for tumor rejection. Third, rejection of the tumor cells via induction of a classic adaptive response would have been expected to result in delayed destruction of tumors after a phase of normal initial growth, which is in contrast with our experimental findings. Thus taken together, although not formally ruled out at the current status of experimental work, enhanced rejection or growth restriction of B16 tumors in partial chimeras via a classical T cell mediated or otherwise adaptive immune response alone does not seem to constitute a likely scenario, suggesting other immune mechanisms as key players.

In fact, it has long been established, that rejection of tumors by the immune system also involves innate mechanisms, executed mainly by macrophages and NK cells [37,38]. With respect to macrophage-mediated antitumor mechanisms, differences between partial chimeras and the syngeneic host are hardly conceivable, since macrophage tumor cell recognition and destruction is not related to strain specificities and has been shown to work even across species barriers [39]. NK cells, on the other hand, recognize their target cells by a lack of inhibitory self structures, namely MHC-I molecules, recognized by a family of clonally distributed NK cell inhibitory receptors [40-42]. Thus, it has been demonstrated, that NK cells are capable of alloreactivity due to mismatched MHC-I molecules [43,44]. However, this phenomenon is not sufficient for rejection of normal tissue grafts, but has been shown to have marked effects on the survival of hematopoietic grafts [45] and the growth of allogeneic tumors [46-48], and thus has led to trials for adoptive transfer of allogeneic NK cells for the treatment of cancer [49-52]. While still a lot remains to be learned about selective NK activity against allogeneic tumors, the current interpretation of these observations is, that activation of NK cell mediated cytotoxicity requires a second stimulus in addition to mismatched or missing MHC-I, provided by stress-induced ligands for activating receptors on NK cells such as the NKG2D-receptor [53,54], which are abundant on many tumor cell types but are absent on most normal tissue cells [55,56].

How might this translate to tumor rejection in the chimeras? B16 tumor cells should be recognized by FVB NK cells, which constitute the vast majority of NK cells in the white skin chimeras, both by their lack of proper matching MHC-I molecules and via recognition of stress markers, whereas normal tissue cells should lack the latter, and should thus be spared. In fact, B16 tumor cells are known targets for NK activity [40], despite some level of MHC-I expression, suggesting that the signals provided by ligation of the inhibitory receptors are overridden by signals from as yet unknown ligands for NK activating receptors, as B16 cells do not express ligands for the NKG2D receptor on NK cells [55]. In addition to NK cell mediated killing of tumor cells, activated NK cells would also provide the appropriate cytokine signals for activation of macrophage antitumor functions [38], starting a concerted innate tumor defense reaction. This may actually explain the enhanced rejection of the B16 tumors in the skin of partial chimeras as compared to the syngeneic host, where no activation of NK cells via mismatched MHC-I molecules can happen. On the other hand, mechanisms of NK allotolerance may also be involved in the differential growth of these tumors in full vs partial chimeras. As demonstrated in an elegant study on mosaic mice, which express a transgenic MHC-I allele in only part of their cells [57], NK cells develop dominant tolerance to cells lacking MHC-I transgene expression. Interestingly however, this form of induced NK allotolerance, which so far is poorly understood at the molecular level, has been shown to be dependent on the continuous presence of tolerizing cells lacking the MHC-I transgene, but to be readily reversible on removal of these cells. Thus, in a chimeric tissue, NK cells would be expected to be dominantly tolerant of missing or mismatched MHC-I molecules, whereas in a non-chimeric tissue NK alloreactivity should be restored on migration of NK cells from chimeric to allogeneic tissues. Based on this notion, NK alloreactivity to the same tumor should be highly dependent on the level of chimerism of the affected organ, which would be in perfect accordance with our experimental findings. In addition, NK-mediated tumor killing may not be operative in fully chimeric tissues, due to the suppressive cytokine environment generated via peripheral CD8^+ ^T_reg _cells [58-60] as discussed above, allowing the tumor to escape also from innate immunity.

## Conclusion

While direct experimental evidence for most of these assumptions is still lacking and will have to await further studies, important conclusions can be drawn from the data at hand.

First, our data suggest that tolerance to self structures in chimeric mice may be maintained primarily by peripheral tolerance mechanisms, potentially also resulting in a reduced level of overall adaptive immunity, as exemplified by the enhanced growth of B16 tumors in chimeric vs syngeneic skin. Still however, these mice are healthy and do not seem to suffer from an increase in spontaneous infections. This suggests that such a reduced level may be well compensated for by other mechanisms of immunity, possibly provided by the innate immune system. Second, and with special respect to tumor immunity, our data also suggest that rejection of tumors by the host immune system is primarily controlled by the local immune milieu of the tumor site, irrespective of the level of systemic adaptive immunity to the very same structures. This comes as quite a surprise and at the same time raises questions about the concept of therapeutic tumor vaccinations. On the other hand, the independence of tumor rejection observed in the non-chimeric skin of otherwise chimeric mice of established central tolerance, as demonstrated by rapid tumor growth at other sites than the skin, suggests that local tumor rejection may be mainly dependent on innate mechanisms, possibly involving NK cells and macrophages. Given this notion and the highly efficient rejection of the B16 tumors in the skin of partial chimeras as compared to syngeneic hosts, one cannot help but wonder whether the natural role of the adaptive immune system during tumor progression is not predominantly a tumor-protective rather than a defensive one, actually hindering the execution of innate and adaptive antitumor mechanisms alike. Future work will thus be devoted to elucidating the relative roles of various components of the immune system for the rejection of tumors in these unique chimeras, as a model for local antitumor immune reactions.

## Competing interests

The authors declare that they have no competing interests.

## Authors' contributions

AYW devised the concept of the present work and performed the experimental work together with EH, except for FACS analysis of immune cells which was performed by LH. Experimental work was performed under the guidance of XY and GS. The manuscript was drafted by AYW and GS. All authors critically reviewed the manuscript and approved its final version.

## Pre-publication history

The pre-publication history for this paper can be accessed here:


